# Amino acid-based formula with synbiotics for cow's milk protein allergy: a real-world study of symptom evolution and quality-of-life outcomes

**DOI:** 10.3389/fped.2026.1864706

**Published:** 2026-07-06

**Authors:** Sebastián Ramiro Soria, Pablo Malagrino, Estefanía Sacconi, Graciela Martin, Nicolás Rovati, Verónica Plante, Florencia Grisel Ursino, María Florencia Biasoli, Natalia Alejandra Petriz, Paula Micone, Eugenia Maciero, Norberto Giglio

**Affiliations:** 1 CEP Pediatric Specialty Clinics, San Miguel de Tucumán, Argentina; 2 Belgrano R Pediatric Medical Center, Ciudad Autónoma de Buenos Aires, Argentina; 3 Uriburu Pediatric Practice, Ciudad Autónoma de Buenos Aires, Argentina; 4 Titiko Ume Private Practice, Corrientes, Argentina; 5 Department of Gastroenterology, 9 de Julio Sanatorium, San Miguel de Tucumán, Argentina; 6 Pediatric Consultation Clinic Dr. Plante, Ciudad Autónoma de Buenos Aires, Argentina; 7 Pediatric Consultation Clinic GastroHepi, Marina Orsi, Ciudad Autónoma de Buenos Aires, Argentina; 8 Pediatric Consultation Clinic Dr. Biasoli, Ciudad Autónoma de Buenos Aires, Argentina; 9 Pediatric Consultation Clinic Plaza, Ciudad Autónoma de Buenos Aires, Argentina

**Keywords:** amino acid formula, cow's milk protein allergy, gastrointestinal symptom, infant nutrition, quality of life, synbiotics

## Abstract

**Background:**

Cow's milk protein allergy (CMPA) is a common early-life food allergy. Evidence from routine care settings may complement randomized trials by describing symptom trajectories in heterogeneous populations.

**Objective:**

This study aimed to describe changes in symptom scores and caregiver-reported outcomes over 28 days following initiation of an amino acid-based formula containing synbiotics in infants with suspected or confirmed CMPA in routine practice.

**Methods:**

This was a prospective, multicenter, observational, single-arm real-world cohort study. Physicians recorded cow’s milk allergy symptom score (CoMiSS) and infant gastrointestinal symptom questionnaire (IGSQ-13) at baseline (Day 1) and Day 28. Caregivers completed the atopic dermatitis patient-oriented score (PO-SCORAD) and quality of life in food allergy-parental burden (FAQL-PB) at baseline and Day 28 and recorded stool, regurgitation, and discomfort at prespecified time points. One-year outcomes (growth and health resource use) were assessed descriptively among participants with available follow-up data.

**Results:**

A total of 71 infants completed the 28-day observation period. Mean CoMiSS decreased from 10.1 (SD 4.6) at baseline to 3.5 (SD 2.2) on Day 28. Median IGSQ-13 and PO-SCORAD scores decreased over 28 days; FAQL-PB scores also decreased. Caregiver-reported stool frequency and regurgitation categories improved over time. One-year outcomes were available for 56 children and are reported descriptively.

**Conclusion:**

In this prospective real-world cohort, symptom scores and caregiver-reported outcomes improved over 28 days following initiation of an amino acid-based formula containing synbiotics in infants managed for suspected/confirmed CMPA. Given the observational nature of the study, these findings should be interpreted cautiously and do not establish causality or comparative effectiveness.

## 1 Introduction

Over the past few years, food allergies have become a growing problem (1), affecting 8% of children in developed countries (2). The causes are thought to be a combination of environmental factors and an underlying genetic susceptibility (3). Among food allergies, cow's milk protein allergy (CMPA) is one of the most frequent allergies during the first years of life (4, 5). In developed countries, the prevalence of CMPA in children under 1 year of life ranges from 0.5% to 3% (6, 7), while its incidence ranges from 2% to 7.5% (4, 8). In breastfed children, treatment of CMPA is based on the exclusion of dairy protein from the mother’s diet, while children who are not being breastfed should receive hypoallergenic formulas, which can be either extensively hydrolyzed protein formulas (EHFs) or amino acid formulas (AAFs) (9–11). Gut microbiota plays a central role in the development and maintenance of oral food tolerance (12–15). The gut microbiota can modulate immune function and therefore plays a role in the development of diseases (16, 18).

In this context, randomized controlled trials (RCTs) have evaluated the use of synbiotics in modulating the immune response associated with food allergies (19, 20). RCTs are designed to evaluate the efficacy of a treatment in a defined and controlled setting, where strict patient inclusion/exclusion criteria allow the selection of the population so that treatment benefits can be derived in a cause-and-effect relationship. While this approach maximizes the internal validity of the study, it also minimizes interindividual variability due to selection bias, implying that the results might have limited external validity. The results obtained with this approach might not be generalizable to the broad spectrum of patients who are likely to use the treatment in everyday real-world clinical practice. To achieve external validity, studies should be conducted with variable and complex populations, such as in real-world evidence studies (RWE). The *National Institute for Health and Care Excellence* defines RWE studies as “evidence generated from the analysis of real-world data” (21).

There is limited RWE to demonstrate the effects of introducing an amino acid formula containing synbiotics in children with cow's milk allergy.

Therefore, the primary objective of this study was to describe the change in clinical symptoms from baseline (Day 1) to Day 28 after initiation of the study formula in routine care. The secondary objectives were to describe changes in infant gastrointestinal symptom questionnaire (IGSQ-13), atopic dermatitis patient-oriented score (PO-SCORAD), and quality of life in food allergy-parental burden (FAQL-PB) over the same period; to describe caregiver-reported stool, regurgitation, and discomfort trajectories for 28 days; and to describe 1-year health resource use among those with available follow-up data.

## 2 Materials and methods

### 2.1 Design and description of the study

**Figure 1:**
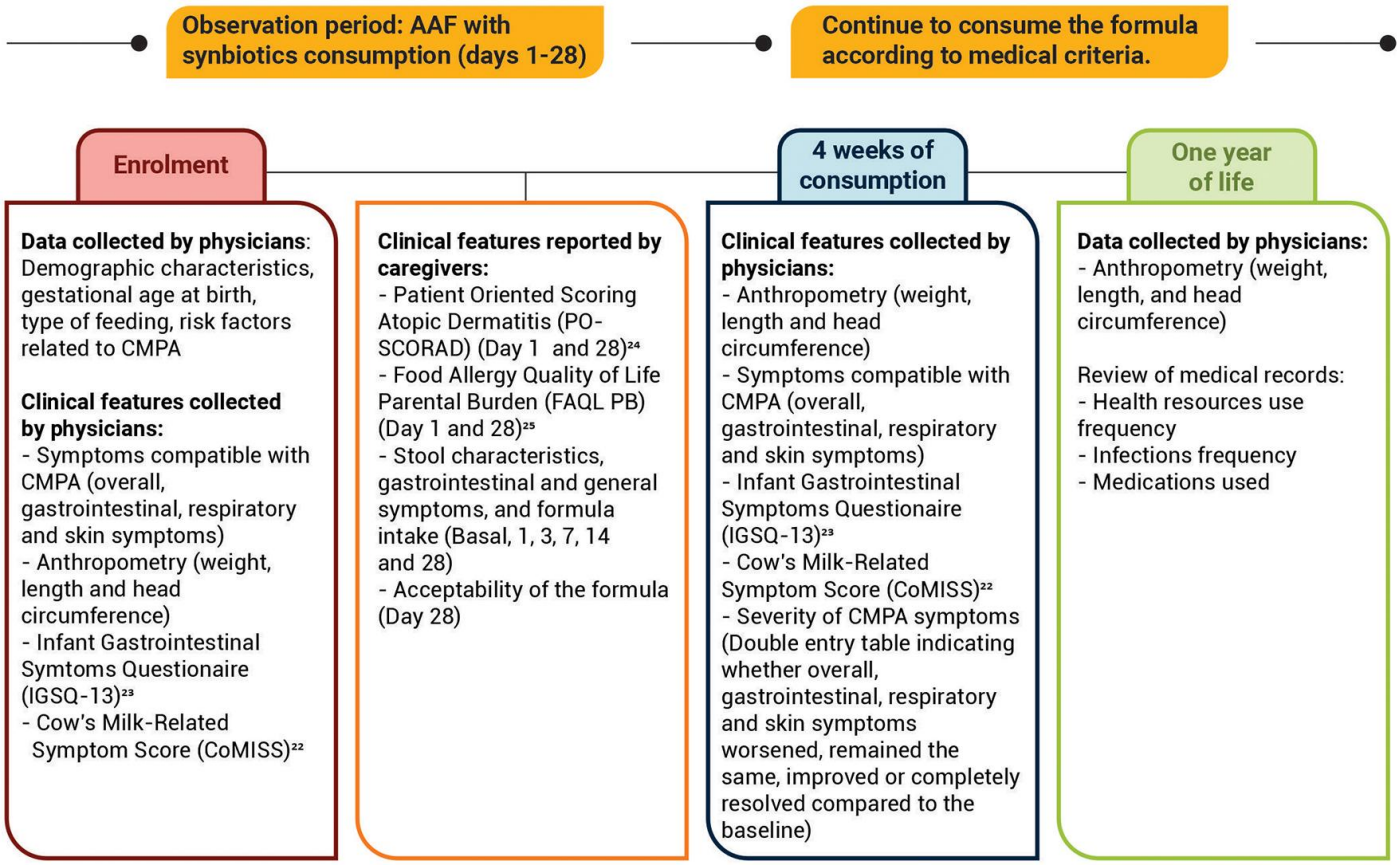
Study design and data collection schedule. The figure summarizes the study timeline, including enrollment, the 28-day observation period with an amino acid-based formula with synbiotics, and follow-up at 1 year of life. It also outlines the variables collected by physicians and caregivers at each time point Adapted from Soria et al. Front Allergy (2023) 4:1265083, under CC BY 4.0.

This was an observational, longitudinal, prospective, and multicenter study based on real-world evidence. The study was carried out in 10 centers located in four provinces of Argentina between May 2022 and May 2023. An overview of the study design and data collection schedule is provided in Figure 1. After enrollment, infants with suspected CMPA received an amino acid–based formula with synbiotics for 28 days. Physicians collected clinical and anthropometric data at baseline and after 4 weeks, while caregivers completed standardized questionnaires assessing gastrointestinal and skin symptoms, formula tolerance, and quality of life. A follow-up assessment was conducted at 1 year of life to record growth, infections, medication use, and health resource utilization.

### 2.2 Outcomes

The main endpoint for the 28-day analysis was symptom evolution between baseline and Day 28 after initiation of the study formula in routine care. Additional outcome measures included changes in cow’s milk allergy symptom score (CoMiSS), IGSQ-13, PO-SCORAD, and FAQL-PB scores over the same period; caregiver-reported trajectories of stool frequency, stool consistency, regurgitation, and digestive discomfort during the 28-day treatment period; formula acceptance; and, among participants with available follow-up data, 1-year outcomes related to growth, infections, medication use, and health resource utilization.

### 2.3 Study population

Infants with confirmed or highly suspected CMPA who had a first indication for an amino acid-based formula (Neocate Syneo®, Nutricia, Liverpool, UK) based on the treating physician’s judgment in routine care, and who were 8 months of age or younger at the time of recruitment, were included. Neocate Syneo® is an AAF with scFOS/lcFOS in a 9:1 ratio and *Bifidobacterium breve* M-16V, designed for the dietary management of CMPA in infants, either as a sole source of nutrition or as a complement to breastfeeding and/or complementary foods. Both the final evaluation and subsequent follow-up of each infant were performed according to the usual practices for patients with suspected CMPA.

### 2.4 Inclusion and exclusion criteria

Patients 8 months of age or younger with confirmed or highly suspected CMPA who had a first indication for an AAF based on the physician's judgment were included. Physicians' criteria were based on international recommendations (10, 11) [malnutrition, urticaria, severe eczema, anaphylaxis, food protein-induced enterocolitis syndrome (FPIES), eosinophilic esophagitis, and multiple allergies] and on extended local practice considering symptoms such as rectal bleeding, risk of growth retardation, risk of residual allergenicity in severe cases, and the need for a safe treatment option to avoid diagnostic delay. Infants who had previously been fed hypoallergenic formulas or who had contraindications to consuming synbiotics (short bowel, parenteral nutrition, postpyloric feeding, central venous catheter, and/or immunocompromise) were excluded. For the 1-year follow-up, all infants who had completed the 28-day initial treatment were included. Children whose data could not be extracted from medical records were excluded from this analysis.

### 2.5 Data collection

Data collection was carried out by physicians and caregivers. Physician-reported outcomes included anthropometry, CoMiSS, and IGSQ-13. Caregiver-reported outcomes included PO-SCORAD, FAQL-PB, stool/regurgitation frequency, and digestive discomfort. As this was an unblinded, single-arm study, caregiver- and clinician-reported outcomes may be affected by expectation, reporting, and observer bias. An overview of the study design and data collection schedule is provided in Figure 1.

One physician: anthropometry, CoMiSS (22) and IGSQ-13 (23) at baseline and after 28 days of treatment. At 1 year of age, anthropometry and retrospective review of medical records were carried out, registering the frequency of attendance to medical services, the rate of infections, and use of medication during the period.

Two caregivers: PO-SCORAD (24) and FAQL-PB (25) were recorded at baseline and after 28 days of treatment. Characteristics and frequency of stools and regurgitation were recorded at baseline and on Days 1, 3, 7, 14, and 28. Stool frequency was recorded by caregivers as the number of bowel movements in the previous 24 h at each time point. Thus, “daily stools” were collected as a whole number (integer value, number of stools per day). For descriptive analyses, stool frequency was categorized as ≤4 vs. ≥5 stools/day. This cut-off was chosen based on clinical judgment to distinguish stool frequencies considered normal in this age group (up to 4 stools/day) from increased stool frequency (≥5 stools/day). Regurgitation frequency was reported by caregivers as the number of regurgitation episodes in the previous 24 h (whole number, episodes/day) and, for analysis, was categorized as ≤3 vs. >3 episodes/day. Digestive discomfort was rated by caregivers for the previous 24 h using four categories (“none,” “slightly,” “some,” “a lot”). For the analyses, we focused on the proportion of children without digestive discomfort (“none”), grouping the other categories together as “with digestive discomfort.” Formula acceptance was recorded on Day 28.

The cutoff values for interpreting the different questionnaires can be seen in Supplementary Table S1.

### 2.6 Statistical analyses

Data reported by physicians and caregivers were collected using Google Forms and analyzed using Stata software. Categorical variables were described as frequencies and percentages, while numerical variables were summarized as mean ± SD or median and interquartile range, as appropriate. Changes in paired outcomes between baseline and Day 28 were assessed using the Wilcoxon signed-rank test. For symptom progression outcomes in which baseline symptom presence was uniform by design, proportions improved/resolved at Day 28 were reported together with exact binomial 95% confidence intervals, and *P*-values were obtained using the exact binomial test. For caregiver-reported repeated measures collected at Days 1, 3, 7, 14, and 28 (stool frequency, stool consistency, regurgitation, and digestive discomfort), overall changes across time were assessed using the Friedman test, followed by paired Wilcoxon tests for comparisons between Day 1 and subsequent time points when appropriate. Exact *P*-values were reported for small samples. A two-sided *P*-value <0.05 was considered statistically significant.

### 2.7 Ethical considerations

Approval from the Independent Ethics Committee for Clinical Pharmacology Trials of the Foundation for Pharmacological and Drug Studies (FEFYM) “Luis María Zieher” was obtained, and the protocol was registered in the computerized registry Platform for Health Research of Buenos Aires (PRIISA.BA). The study was carried out following Good Clinical Practice and the Declaration of Helsinki. All patientś parents signed written informed consent.

## 3 Results

### 3.1 Study population

**Figure 2:**
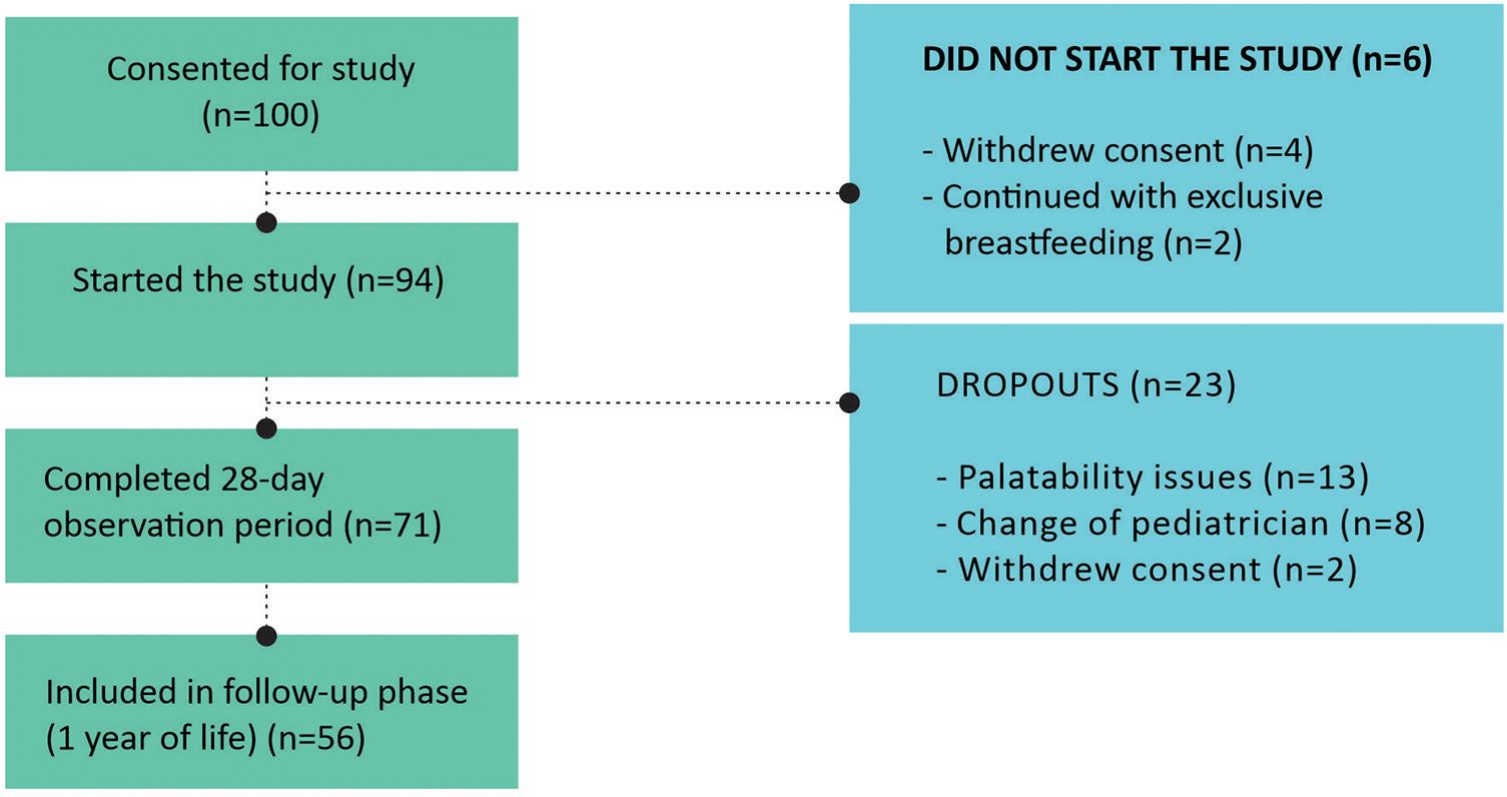
Study flow from enrollment to 1-year follow-up. Of the 100 infants who consented to participate, 94 started the study, 71 completed the 28-day observation period, and 56 were included in the follow-up phase at 1 year of life. Reasons for not starting the study and for dropout are shown in the figure.

A total of 100 infants were enrolled after parental consent was obtained; 94 started the study, and 71 completed the 28-day observation period. The main reasons for discontinuation were palatability issues, change of pediatrician, and withdrawal of consent. Two infants whose parents had provided informed consent did not initiate the study formula and were therefore not included in the observational cohort. In both cases, exclusive breastfeeding under a maternal exclusion diet was established before the planned start of formula administration, and the infants were not started on the study formula. At the 1-year follow-up, outcome data were available for 56 children among the 71 who completed the 28-day period; the remaining 15 children did not attend the 1-year visit and were considered lost to follow-up. The recruitment flow is presented in Figure 2.

The demographic characteristics of the population, the family history of atopy in biological relatives, and the initial symptoms compatible with CMPA are summarized in Tables 1–3, respectively. The high proportions of C-section, premature births, and family history of atopy are highlighted. All subjects presented gastrointestinal symptoms.

**Table 1:** Baseline demographic characteristics, perinatal history, and feeding type at study onset (*n* = 71).

Characteristics	n (%)
Sex^a^	
Female	37 (52)
Male	34 (48)
Age at admission (months)^b^	2.9 ± 1.95 (0–8)
Premature birth (gestational age <37 weeks)^a^	16 (23.5)
Route of delivery^a^	
Vaginal	13 (19)
C-section	58 (81)
Type of feeding at study onset^a^	
Mixed feeding	62 (87)
Exclusive formula	9 (13)

a *n* (%).

b Mean ± SD (range).

**Table 2:** Family history of atopy in biological relatives (*n* = 71).

Family history of atopy (*n* = 71)	*n* (%)
None	36 (50)
Two or more relatives	12 (17)
Mother	12 (17)
Father	4 (5)
Siblings	7 (11)

**Table 3:** Baseline symptom patterns compatible with CMPA (*n* = 71).

Symptom pattern compatible with CMPA	*n* (%)
Exclusive gastrointestinal	47 (66)
Dermatological + gastrointestinal	19 (27)
Dermatological + respiratory + gastrointestinal	4 (6)
Respiratory + gastrointestinal	1 (1)

CMPA, cow’s milk protein allergy.

a Rectal bleeding was present in 49% of patients (*n* = 35).

### 3.2 Impact of using an AAF with synbiotics over 28 days

Table 4 shows the changes in symptoms recorded by the physicians after 28 days of treatment. The initial symptoms resolved or disappeared in 95% or more of the patients, and none of the patients experienced a worsening of the onset symptoms.

**Table 4:** Symptom progression after 28 days of treatment with an amino acid-based formula containing synbiotics.

Outcome	Improved or resolved, *n*/*N* (%) [95% CI]	Stayed the same, *n*/*N* (%) [95% CI]	*P*-value
Gastrointestinal symptoms (*n* = 71)	68/71 (95.8%) [95% CI: 88–99]	3/71 (4.2%) [95% CI: 0.8–11]	<0.0001
Dermatological symptoms (*n* = 23)	21/23 (91.3%) [95% CI: 77–99]	2/23 (8.7%) [95% CI: 0.1–22]	<0.0001
Respiratory symptoms (*n* = 5)	5/5 (100%) [95% CI: 48–100]	0/5 (0.0%) [95% CI: 0–52]	<0.0625
Overall symptoms (*n* = 71)	68/71 (95.8%) [95% CI: 88–99]	3/71 (4.2%) [95% CI: 0.8–11]	<0.0001

CI, confidence interval.

Data were reported by physicians. Table 4 presents the proportion of infants whose symptoms improved or resolved by Day 28, with exact binomial 95% CIs and *P*-values from the exact binomial test.

Mean CoMiSS recorded by physicians decreased from 10.1 ± 4.6 before treatment (Day 1) to 3.5 ± 2.2 after treatment (Day 28). At baseline, 34 patients had values greater than or equal to 10. After 28 days of treatment, no patient exceeded 10 points.

Additional repeated-measures analyses across Days 1, 3, 7, 14, and 28 for caregiver-reported stool frequency, stool consistency, regurgitation, and digestive discomfort are presented in Supplementary Table S4.

**Figure 3:**
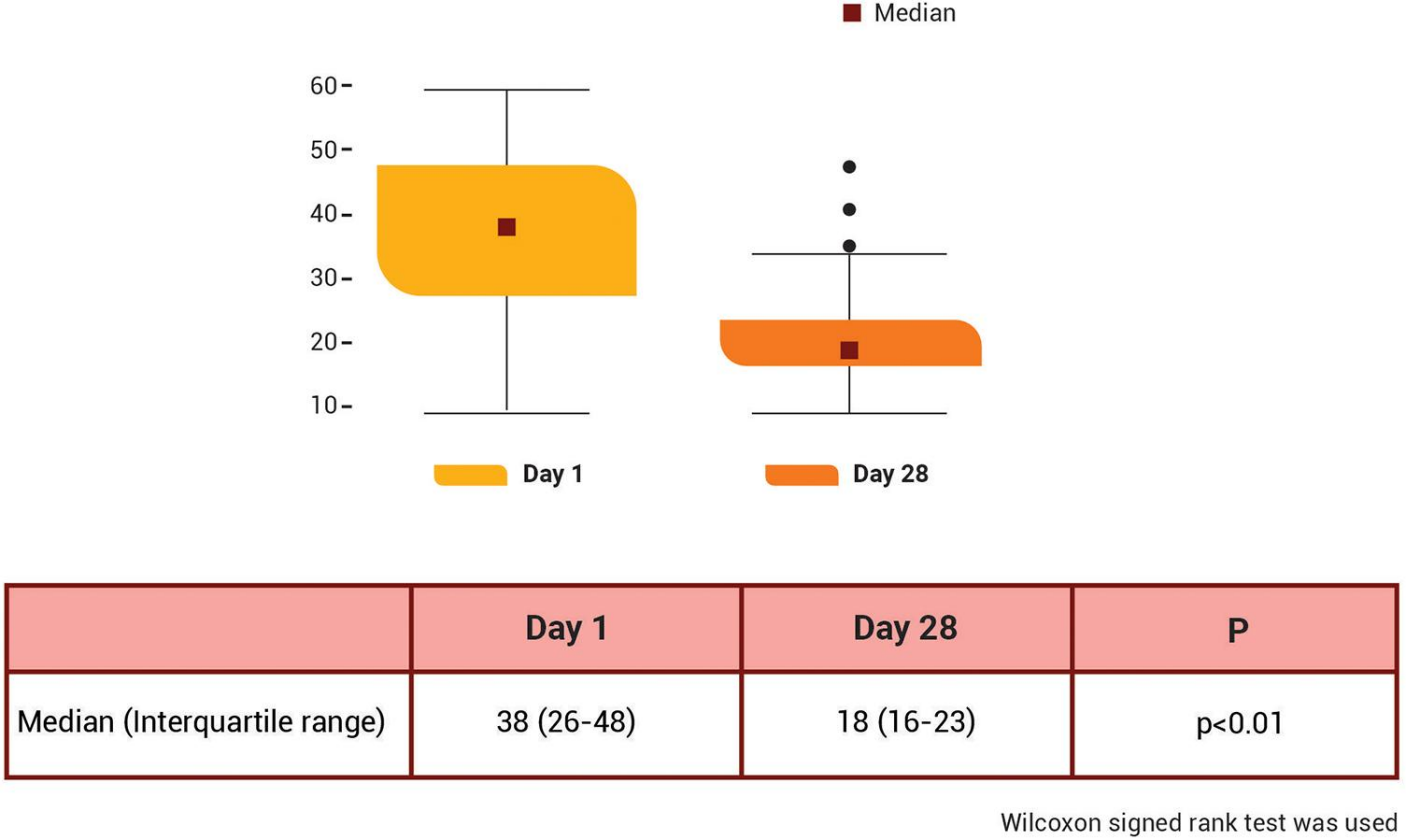
Infant gastrointestinal symptoms questionnaire (IGSQ-13) score at baseline and Day 28. Median (interquartile range) IGSQ-13 scores decreased from 38 (26–48) at baseline to 18 (16–23) at Day 28 (*P* < 0.01). The Wilcoxon signed-rank test was used.

IGSQ-13 results, as assessed by physicians, are presented in Figure 3. A statistically significant improvement in gastrointestinal symptoms was observed after 28 days of treatment with an AAF containing synbiotics.

**Figure 4:**
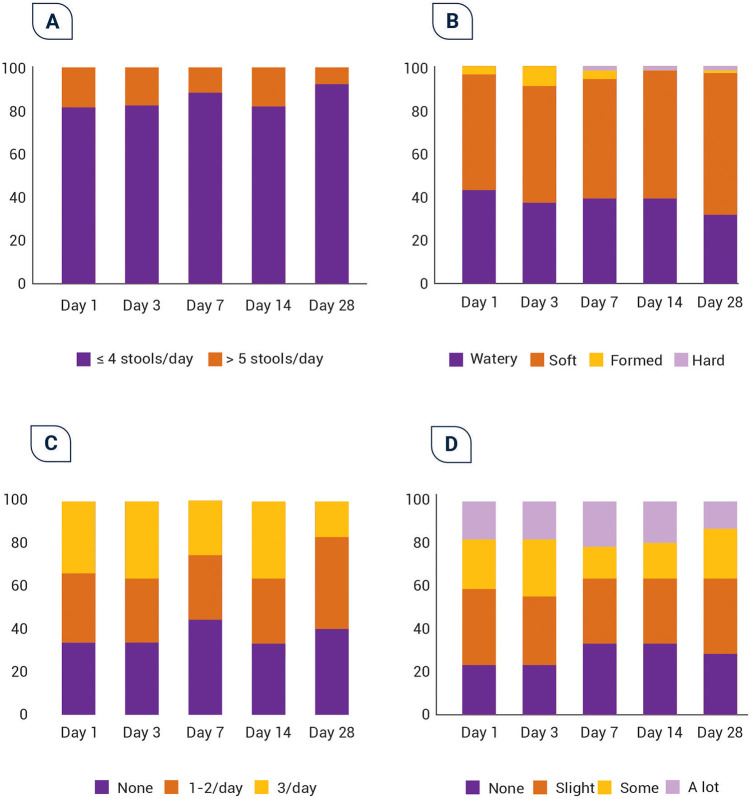
Caregiver-reported changes in gastrointestinal symptoms during treatment. **(A)** Changes in stool frequency during treatment. **(B)** Changes in stool consistency during treatment. **(C)** Changes in regurgitation frequency during treatment. **(D)** Changes in visual signs of discomfort attributed to digestive symptoms according to caregivers.

Changes in stool frequency and consistency, as well as in the frequency of regurgitation, according to the information provided by caregivers during the 28 days of treatment, are presented in Figure 4. The proportion of patients with fewer than four daily stools increased from 79% (Day 1) to 90% (Day 28) (*P* = 0.03). In that same period, the proportion of children with more than three episodes of regurgitation per day decreased from 32% to 17% (*P* = 0.01). The proportion of children without daily episodes of regurgitation increased from 37% to 52% (*P* = 0.03). The proportion of children without symptoms of discomfort attributed to digestive symptoms increased from 25% to 39% in the same period (*P* = 0.03).

**Figure 5:**
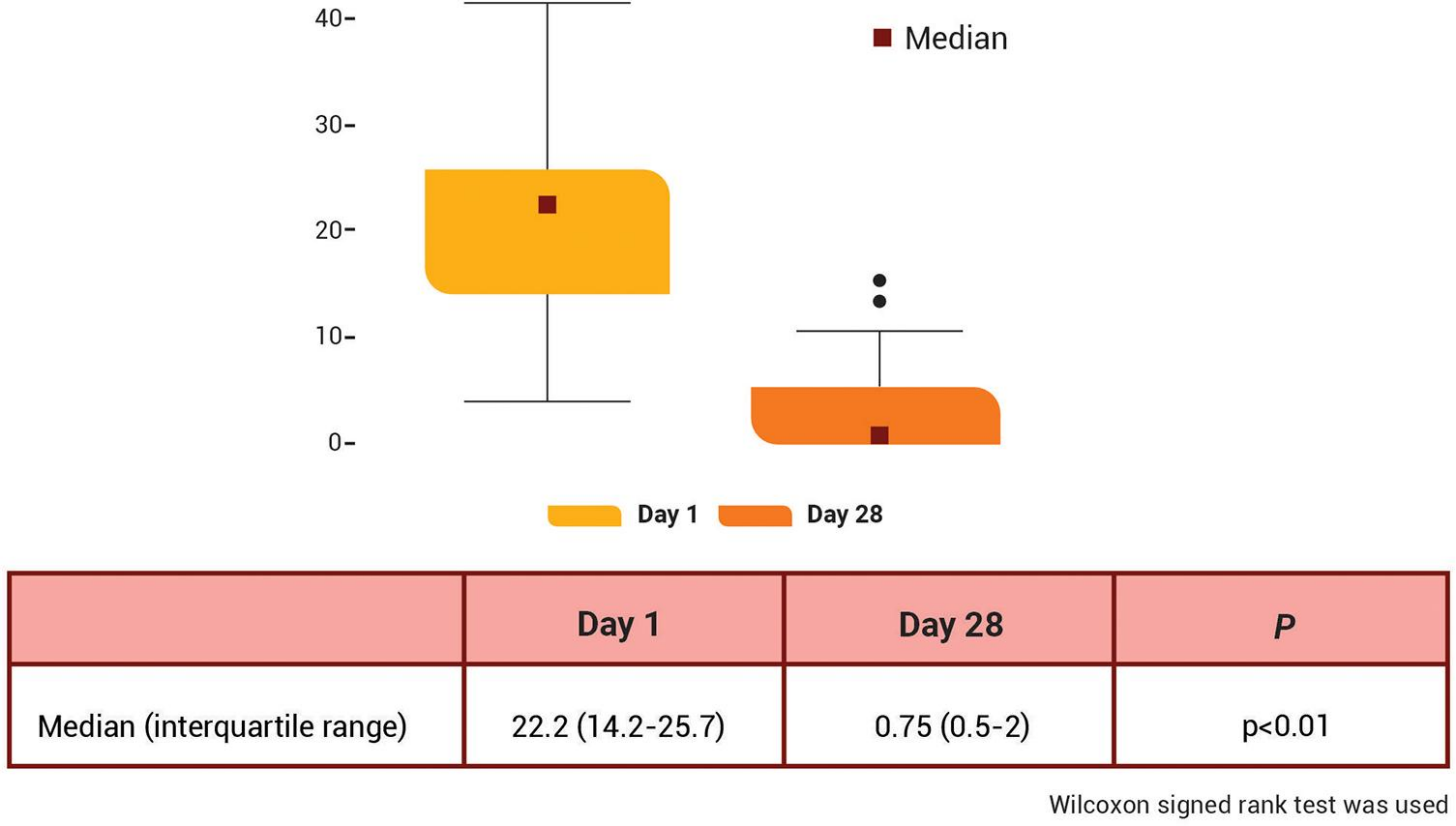
Change in patient-oriented SCORing atopic dermatitis (PO-SCORAD). Median (interquartile range) PO-SCORAD values decreased from 22.2 (14.2–25.7) at baseline to 0.75 (0.5–2) at Day 28 (*P* < 0.01). The Wilcoxon signed-rank test was used.

Changes in PO-SCORAD from baseline to Day 28 are presented in Figure 5.

**Figure 6:**
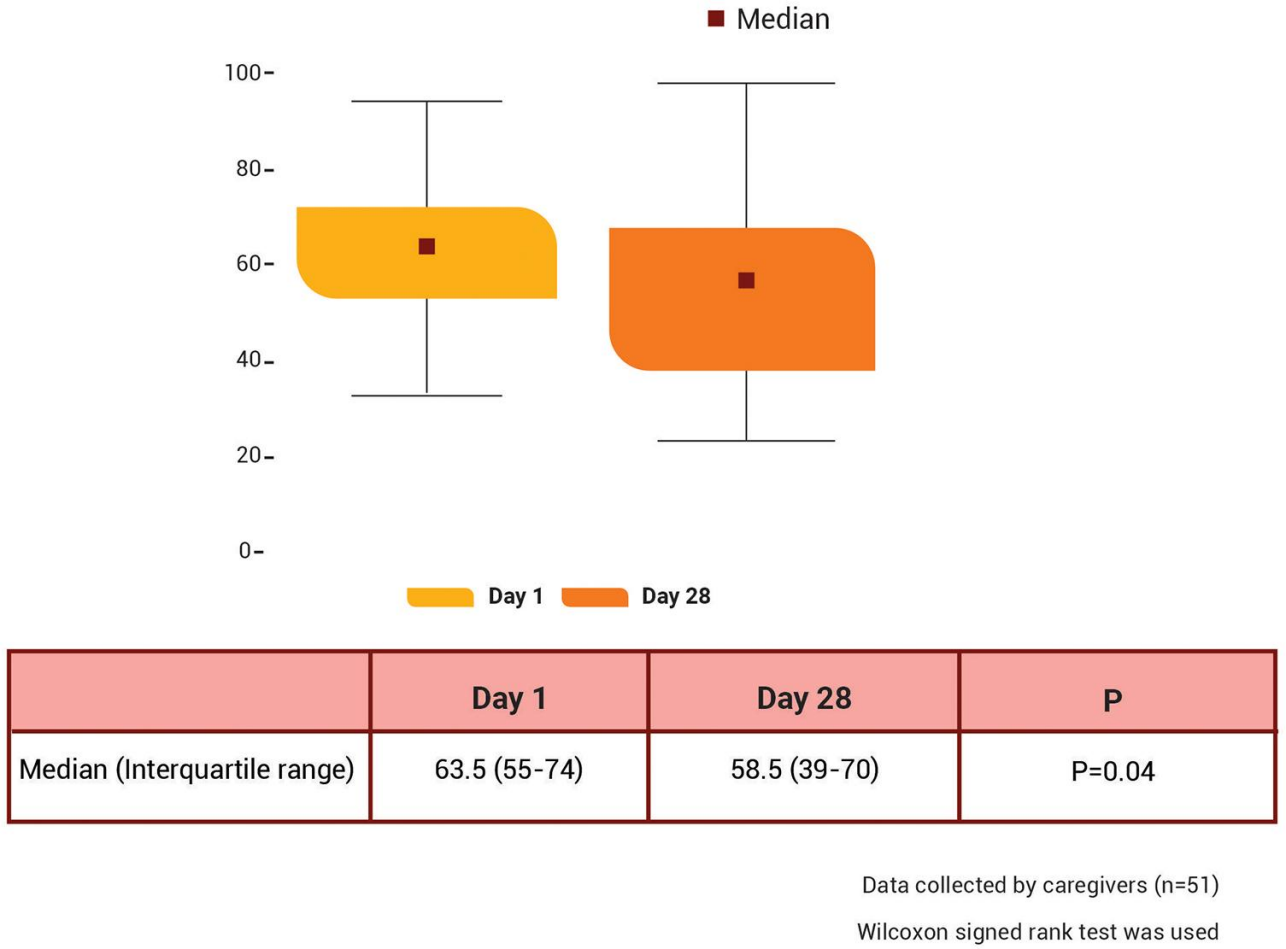
Change in the food allergy quality of life-parental burden (FAQL-PB). Median (interquartile range) FAQL-PB scores decreased from 63.5 (55–74) at baseline to 58.5 (39–70) at Day 28 (*P* = 0.04). Data were collected by caregivers (*n* = 51). The Wilcoxon signed-rank test was used.

FAQL-PB scores over the same period are presented in Figure 6. While these changes were statistically significant, their clinical relevance may vary according to the individual patient and family context.

Supplementary Table S2 [weight, height, and head circumference (HC) in infants younger than 3 months] and Supplementary Table S3 (weight, height, and HC in infants older than 3 months) present anthropometric data showing changes consistent with age-expected growth in infants treated with an AAF containing synbiotics.

In relation to basic requirements for adequate adherence to treatment (*n* = 56), 100% of caregivers reported that the formula was easy to prepare, and 62% of the patients seemed to enjoy it.

### 3.3 Impact of the use of an AAF with synbiotics at 1 year of age

After 1 year of life, the number of visits to the emergency medical department and the number of hospitalizations were retrospectively evaluated. Among the children, 54.7% never attended emergency consultations, 27.1% attended the emergency service fewer than five times, and 18.2% attended five or more times, while only 16.3% had hospitalizations during the first year of life that were not related to CMPA. A total of 24% of patients required the use of antibiotics once, while only 7% of patients required antiallergic medication.

One-year outcomes were assessed retrospectively among participants with available follow-up data (*n* = 56). Because follow-up was incomplete, these findings are descriptive and may be influenced by attrition/selection bias; they should not be interpreted as representative of the full cohort.

## 4 Discussion

The indication of AAF during the elimination phase represents a strategy aimed at minimizing the risk of persistent allergic symptoms associated with EHF in approximately 10% of patients with CMPA (12, 22, 23). This approach enhances diagnostic accuracy, accelerates the identification of non-responders, and reduces the psychosocial and nutritional burden on patients and their families. Furthermore, the adoption of an AAF diagnostic protocol has been associated with a shorter duration of symptomatic periods and earlier initiation of effective treatment (12, 22), improvement in clinical outcomes, and a reduction in direct healthcare costs through the avoidance of prolonged diagnostic processes and unnecessary therapeutic interventions (24, 25).

Several physicians consider that initiating the elimination diet with AAF is cost-effective (24, 25) and that proper management of CMPA implies the complete elimination of milk proteins from the diet, advising mothers to exclude milk proteins while maintaining breastfeeding whenever possible, and to use an AAF only when breastfeeding is not feasible or cannot be sustained.

At baseline, the average CoMiSS score was 10.1 ± 4.6 on Day 1, not as high as expected. This can be explained by the fact that many children were breastfed and their mothers reported being on an exclusion diet before receiving AAF.

The high rate of C-sections, prematurity, and family history of atopy observed in the population recruited for the study is consistent with a population of infants with high suspicion of CMPA, since all are known predisposing factors (26, 27). C-section birth interferes with the homeostasis of the gut microbiota and is a determinant of dysbiosis (28–32), which is one of the main culprits in the increased incidence of allergic disorders.

The subjects enrolled in this study showed digestive, dermatological, and respiratory symptoms. While it has been reported that generally between 50% and 60% of CMPA symptoms are gastrointestinal (22), 100% of the subjects included in this study had these symptoms, and 49% of them presented rectal bleeding, a common condition for referral to the specialist. This bias is possibly because 90% of the researchers were pediatric gastroenterologists.

Over the 28-day observation period, we observed improvements in symptoms and questionnaire scores following the introduction of the study formula. These observations are consistent with prior randomized trials evaluating similar formulations (19, 20, 37). These improvements could also be evidenced by the good evolution of CoMiSS and PO-SCORAD.

CoMiSS is a tool that integrates information related to general, dermatological, gastrointestinal, and respiratory manifestations. A score greater than or equal to 12 (22) was usually considered to be indicative of CMPA, although in the new updates, it is suggested to reduce this value to a score ≥10 (34), thus improving sensitivity. Other studies (35) indicate that the best sensitivity/specificity ratio would be achieved with cut-off points between 5 and 6.

When interpreting the results of the CoMiSS in this study, it is important to consider that 87% of the patients continued to breastfeed when incorporating the elemental formula. As the mothers were already on an exclusion diet, the CoMiSS on Day 1 is likely to show a lower value than expected. Also, rectal bleeding was frequent in our cohort and is not captured within CoMiSS, which may limit its sensitivity for non-IgE-mediated phenotypes. Given the real-world setting and the lack of systematic oral food challenge, some degree of phenotype heterogeneity and potential misclassification, including presentations compatible with food protein-induced allergic proctocolitis, cannot be excluded. Despite these baseline values, a significant decrease in the score could be seen on Day 28 of treatment. These results are in line with similar reports in EHF-treated CMPA infants treated with oligosaccharides and *Bifidobacterium breve* M-16V (33, 36, 42).

The evolution of dermatological symptoms was studied with the PO-SCORAD (24). Symptoms are considered mild if the score is below 25, moderate if it is between 25 and 50, and severe when the score exceeds 50. In the present study, the patients who entered the protocol had mild dermatological involvement and, after the intervention, a reduction in the PO-SCORAD was observed in 95% of the patients. This significant drop in median PO-SCORAD is in line with reports in infants with CMPA treated with AAF with scFOS/lcFOS and *Bifidobacterium breve* M-16V (36).

Treatment of infants with an AAF with synbiotics decreased both stool and regurgitation frequency, improved stool consistency, and reduced infant discomfort attributed by caregivers to digestive symptoms. These results are similar to those obtained in a multicenter, double-blind, randomized controlled study in patients diagnosed with CMPA who received AAF, and can be explained by the ability of synbiotics to improve symptoms related to dysbiosis (15). Consistent with the results shown in this study, a real-world evidence study showed that infants with suspected CMPA treated with an extensively hydrolyzed formula with the addition of synbiotics (*Bifidobacterium breve* M-16V and scFOS/lcFOS [9:1]) had decreased frequency of regurgitation and stools, improved stool consistency, and reduced infant discomfort (36).

Another aspect that should be considered in patients with CMPA is the impact on the quality of life of infants and their caregivers (38, 39). Parents or caregivers of infants with food allergies report a lower quality of life, as well as high levels of anxiety and stress, due to the social and emotional impact they suffer (40). FAQL-PB scores decreased over 28 days, suggesting a reduction in parental burden during the early management period (36).

At 1 year of age, the patients' medical history was retrospectively evaluated to describe the use of health resources by infants with high suspicion of CMPA.

The low rate of on-call visits, hospitalizations, infections, and medication use is in line with randomized trials and meta-analyses in which the use of AAF with synbiotics has been associated with a low prevalence of infections and antimicrobial use (17, 43) and a low rate of hospitalizations (40, 41). In a recent RWE study in children with CMPA treated with extensively hydrolyzed formula and synbiotics, 52% of the infants did not require any consultation with the emergency service until 1 year of life, and 42.3% of the infants did not require pharmacological medication during follow-up (36).

This study is one of the first to evaluate the progression of CMPA symptoms treated with an AAF with synbiotics, with a significant sample and in a real-world setting. This type of study allows us to assess the effectiveness of the intervention in the real world, considering interindividual variability and the complexity of each clinical case. Real-life studies offer a powerful tool to complement clinical trial evidence and improve healthcare professionals' decisions.

One inherent limitation of this design is the potential for bias and confounding factors due to the lack of a control group. In addition, several outcomes relied on caregiver report, and some on clinician judgment, in an unblinded setting, which increases susceptibility to expectation, reporting, and observer bias. In addition, diagnostic confirmation by oral food challenge was not systematically performed, which may introduce diagnostic misclassification and phenotype heterogeneity.

In addition, these results cannot be extrapolated to other types of synbiotic combinations, since they are specific to the combination of scFOS/lcFOS and *Bifidobacterium breve* M-16V.

## 5 Conclusion

In this prospective real-world cohort of infants with suspected or confirmed CMPA managed with an amino acid-based formula containing synbiotics, symptom scores and caregiver-reported outcomes improved over 28 days, and growth parameters were consistent with age-expected trajectories.

## Data Availability

The raw data supporting the conclusions of this article will be made available by the authors, without undue reservation.
