# Supplementary material for: Amino acid-based formula with synbiotics for cow's milk protein allergy: a real-world study of symptom evolution and quality-of-life outcomes
Source: Front Pediatr. 2026 Jul 6;14:1864706. doi: 10.3389/fped.2026.1864706 (PMC13381462; doi:10.3389/fped.2026.1864706)
Supplement: Supplementary file 1 [file Table1.docx]

Supplementary Table S1. Questionnaire interpretation and cut-off point

| **Questionnaire** | **Description** | **Score range** | **Interpretation** |
| --- | --- | --- | --- |
| COMISS^15^  (The Cow’s Milk-related Symptom Score) | Evaluates five key areas: crying, regurgitation, stool consistency, and skin and respiratory symptoms. | 0–33 | A score of 10 or more suggests the infant may benefit from further assessment for CMPA. |
| IGSQ-13^16^  (The Infant Gastrointestinal Symptom Questionnaire) | Evaluates the severity of gastrointestinal (GI) symptoms in infants by assessing parents’ observations of their infant’s GI-related signs and symptoms over the previous week. It includes 13 questions across five domains: stooling, spitting up/vomiting, flatulence, crying, and fussiness. Each item is scored on a scale from 1 to 5, with higher scores indicating more severe symptoms. | 13–65 | 13: No gastrointestinal distress; 65: Extreme gastrointestinal distress. |
| PO-SCORAD^17^ (Patient-Oriented SCORing Atopic Dermatitis) | Evaluates the severity of eczema through patient/caregiver self-assessment. It combines the extent of skin affected, the intensity of symptoms such as redness and itching, and the impact on sleep. | 0–103 | 0: No symptoms; 103: Extreme symptoms. |
| FAQL-PB ^18^  (Food Allergy Quality of Life – Parental Burden) | Assesses the degree to which a child’s food allergy affects the caregiver’s life, including emotional well-being and daily activities. It consists of 17 questions rated on a 7-point Likert scale, where higher scores indicate a greater burden on the caregiver. | 17–119 | Higher FAQL-PB scores indicate a greater impact of the child’s food allergy on the caregiver’s quality of life and a greater need for support and resources. |
