# Supplementary material for: Amino acid-based formula with synbiotics for cow's milk protein allergy: a real-world study of symptom evolution and quality-of-life outcomes
Source: Front Pediatr. 2026 Jul 6;14:1864706. doi: 10.3389/fped.2026.1864706 (PMC13381462; doi:10.3389/fped.2026.1864706)
Supplement: Supplementary file 2 [file Table2.docx]

Supplementary Table S2. Growth in infants younger than 3 months (n=45).

| **Measure** | **Group** | **Day 1 mean ± SD** | **Day 28 mean ± SD** | **Mean difference (range)** |
| --- | --- | --- | --- | --- |
| **Weight (g)** | | | | |
| Weight | Boys (23) | 4,644 ± 1,401 | 5,847 ± 1,242 | 1,203 (360–2,400) |
| Weight | Girls (22) | 4,699 ± 1,070 | 5,535 ± 976 | 836 (400–1,250) |
| **Length (cm)** | | | | |
| Length | Boys (23) | 55.4 ± 5.0 | 60.0 ± 4.7 | 4.5 (1–7) |
| Length | Girls (22) | 55.0 ± 5.1 | 58.0 ± 4.4 | 3.1 (1–7) |
| **Head circumference (cm)** | | | | |
| Head circumference | Boys (23) | 38.6 ± 2.3 | 40.8 ± 2.4 | 2.1 (1–8) |
| Head circumference | Girls (22) | 38.1 ± 2.3 | 39.8 ± 2.1 | 1.7 (1–8) |
