# Supplementary material for: Amino acid-based formula with synbiotics for cow's milk protein allergy: a real-world study of symptom evolution and quality-of-life outcomes
Source: Front Pediatr. 2026 Jul 6;14:1864706. doi: 10.3389/fped.2026.1864706 (PMC13381462; doi:10.3389/fped.2026.1864706)
Supplement: Supplementary file 3 [file Table3.docx]

Supplementary Table S3. Growth in infants older than 3 months (n=23).

| **Measure** | **Group** | **Day 1 mean ± SD** | **Day 28 mean ± SD** | **Mean difference (range)** |
| --- | --- | --- | --- | --- |
| **Weight (g)** | | | | |
| Weight | Boys (12) | 7,354 ± 912 | 7,879 ± 781 | 525 (200–1,200) |
| Weight | Girls (11) | 5,918 ± 1,159 | 6,239 ± 1,295 | 375 (180–850) |
| **Length (cm)** | | | | |
| Length | Boys (12) | 64.5 ± 4.6 | 67.3 ± 3.0 | 2.9 (0–9) |
| Length | Girls (11) | 60.0 ± 4.0 | 62.1 ± 3.8 | 2.0 (1–3) |
| **Head circumference (cm)** | | | | |
| Head circumference | Boys (12) | 42.5 ± 1.1 | 43.8 ± 1.2 | 1.2 (0–4) |
| Head circumference | Girls (11) | 40.0 ± 2.1 | 42.9 ± 0.8 | 2.7 (1–9) |
