# Supplementary material for: Amino acid-based formula with synbiotics for cow's milk protein allergy: a real-world study of symptom evolution and quality-of-life outcomes
Source: Front Pediatr. 2026 Jul 6;14:1864706. doi: 10.3389/fped.2026.1864706 (PMC13381462; doi:10.3389/fped.2026.1864706)
Supplement: Supplementary file 4 [file Table4.docx]

Supplementary Table S4. Repeated-measures analysis of caregiver-reported stool frequency, stool consistency, regurgitation, and digestive discomfort from Day 1 to Day 28.

A. Stool frequency

| **Category** | **Day 1** | **Day 3** | **Day 7** | **Day 14** | **Day 28** |
| --- | --- | --- | --- | --- | --- |
| ≤4 stools/day | 49 (79.0%) | 53 (81.5%) | 56 (86.2%) | 52 (82.5%) | 56 (90.3%) |
| ≥5 stools/day | 13 (21.0%) | 12 (18.5%) | 9 (13.8%) | 11 (17.5%) | 6 (9.7%) |

Overall Friedman test p=0.248

Wilcoxon paired comparison (Day 1 vs Day 28) p=0.065

Interpretation: No statistically significant differences were observed. A non-significant trend was noted between Day 1 and Day 28.

B. Stool consistency

| **Category** | **Day 1** | **Day 3** | **Day 7** | **Day 14** | **Day 28** |
| --- | --- | --- | --- | --- | --- |
| Formed | 1 (1.9%) | 5 (8.5%) | 1 (1.7%) | 1 (1.8%) | 2 (3.6%) |
| Soft | 31 (57.4%) | 35 (59.3%) | 36 (60.0%) | 35 (61.4%) | 38 (67.9%) |
| Watery | 22 (40.7%) | 19 (32.2%) | 21 (35.0%) | 20 (35.1%) | 15 (26.8%) |
| Hard | 0 (0.0%) | 0 (0.0%) | 2 (3.3%) | 1 (1.8%) | 1 (1.8%) |

Overall Friedman test p=0.599

Wilcoxon paired comparison (Day 1 vs Day 28) p=0.074

Interpretation: No statistically significant differences were observed. A non-significant trend was noted between Day 1 and Day 28.

C. Regurgitation frequency

| **Category** | **Day 1** | **Day 3** | **Day 7** | **Day 14** | **Day 28** |
| --- | --- | --- | --- | --- | --- |
| None | 23 (37.1%) | 25 (38.5%) | 29 (44.6%) | 24 (38.1%) | 32 (51.6%) |
| 1–2 episodes/day | 19 (30.6%) | 19 (29.2%) | 19 (29.2%) | 18 (28.6%) | 19 (30.6%) |
| ≥3 episodes/day | 20 (32.3%) | 21 (32.3%) | 17 (26.2%) | 21 (33.3%) | 11 (17.7%) |

Overall Friedman test p=0.136

Wilcoxon paired comparison (Day 1 vs Day 28) p=0.017

Interpretation: A statistically significant difference was observed between Day 1 and Day 28.

D. Digestive discomfort

| **Category** | **Day 1** | **Day 3** | **Day 7** | **Day 14** | **Day 28** |
| --- | --- | --- | --- | --- | --- |
| None | 16 (25.8%) | 20 (30.8%) | 23 (35.4%) | 29 (46.0%) | 32 (51.6%) |
| Mild | 21 (33.9%) | 25 (38.5%) | 22 (33.8%) | 18 (28.6%) | 14 (22.6%) |
| Some | 14 (22.6%) | 17 (26.2%) | 18 (27.7%) | 15 (23.8%) | 12 (19.4%) |
| A lot | 11 (17.7%) | 3 (4.6%) | 2 (3.1%) | 1 (1.6%) | 4 (6.5%) |

Overall Friedman test p=0.004

Wilcoxon paired comparison (Day 1 vs Day 14) p=0.001

Wilcoxon paired comparison (Day 1 vs Day 28) p=0.004

Interpretation: Statistically significant overall changes were observed, with significant differences emerging from Day 14 onward.
